# The chloroplast genome of aromatic plants *Cinnamomum burmanni* (Lauraceae)

**DOI:** 10.1080/23802359.2019.1676677

**Published:** 2019-10-16

**Authors:** Yanping Yang, Yu Song, Peiyao Xin

**Affiliations:** a Key Laboratory of Forest Resources Conservation and Utilization in the Southwest Mountains of China Ministry of Education, Southwest Forestry University, Kunming, PR China;; b Center for Integrative Conservation, Xishuangbanna Tropical Botanical Garden, Chinese Academy of Sciences, Yunnan, PR China

**Keywords:** *Cinnamomum*, chloroplast, phylogenetic analysis

## Abstract

*Cinnamomum burmanni* (Nees et T. Nees) Blume is a valuable aromatic timber tree of the genus *Cinnamomum* Tree in the family Lauraceae. To better determine its phylogenetic location with respect to the other *Cinnamomum* species, the complete chloroplast genome of *C. burmanni* was sequenced. The total chloroplast genome size is 152,775 bp, consisting of a pair of inverted repeats (IRa/b) with a length of 20,092 bp separated by a large single-copy region (LSC) and a small single-copy region (SSC) which are 93,687 and 18,903 bp, respectively. The overall GC content of the cp genome is 39.1%. Further, maximum-likelihood phylogenetic analysis with K3Pu + F+I model was performed using eleven complete plastomes of the Lauraceae, which revealed that *C. burmanni* is closely related to *C. verum*.

*Cinnamomum burmanni* (Nees et T. Nees) Blume is an economically important evergreen tree that mainly distributed in the south of the Yangtze River in China, India, Indonesia, Myanmar, Philippines, and Vietnam (http://foc.iplant.cn/). The volatile aromatic oil isolated from the stems and leaves of *C. burmanni* are rich in eucalyptol (Liu et al. 2007; Li et al. 2016) and thus represent important woody aromatic plants in the genus *Cinnamomum* (Kumar and Kumari 2019). But currently, there is no clear phylogeny for the *Cinnamomum*. To determine the phylogenetic location of *C*. *burmanni* with respect to the other *Cinnamomum* species (Song et al. 2019), the complete chloroplast genome of *C*. *burmanni* was used to reconstruct a phylogenetic tree based on high throughput sequencing approaches.

Intact, fresh and young leaves of *C*. *burmanni* were obtained from the Xishuangbanna Tropical Botanical Garden (21.9°N, 101.3°E; 549 m above sea level) for genomic DNA extraction (Doyle and Dickson^1987^). The specimens were deposited at the Biodiversity Research Group of XTBG (Accession Number: XTBG-BGR-SY34687). The whole plastid genome was sequenced following Yang et al. (2014), and their 11 universal primer pairs were used to perform long-range PCR for next-generation sequencing. The contigs were aligned using the publicly available plastid genome of *Cinnamomum chago* (Accession Number: LAU00078) (Chen et al. 2019) and annotated in Geneious 4.8.

The chloroplast genome of *C. burmanni* (LAU00110) with a length of 152,775 bp, was the largest of the 11 reported cp genome of *Cinnamomum*, was 10 bp larger than that of *C. verum* (152,765 bp, KY635878) and was 101 bp smaller than that of *C*. *micranthum* (152,674 bp, KT833081). The complete cp genome of *C. burmanni* composed of a large single-copy (LSC) region of 93,687 bp, a small single-copy (SSC) region of 18,903 bp, and a pair of inverted repeats (IRa/b) of 20,092 bp. The overall GC content is 39.1% (LSC, 38%; SSC, 33.8%; IR, 44.4%).

In order to confirm the evolutionary relationship between *C. burmanni* and other species with published plastomes in *Cinnamomum,* we reconstructed a phylogenetic tree (Figure 1) based on 10 published plastid genome sequences of the Lauraceae. *Nectandra angustifolia* (Accession Number: MF939340) was treated as an out-group (Song et al. 2017). A maximum-likelihood (ML) analysis based on the K3Pu + F+I model was performed with iqtree version 1.6.7 programme using 1000 bootstrap replicates (Figure 1) (Nguyen et al. 2015). The phylogenetic tree reveals that 68–100% bootstrap values at each node supported that *Cinnamomum* species can be divided into two clades, and sisterhood of *C. burmanni* and *C. verum*, followed by *C. chago* in the same clade.

**Figure 1.**
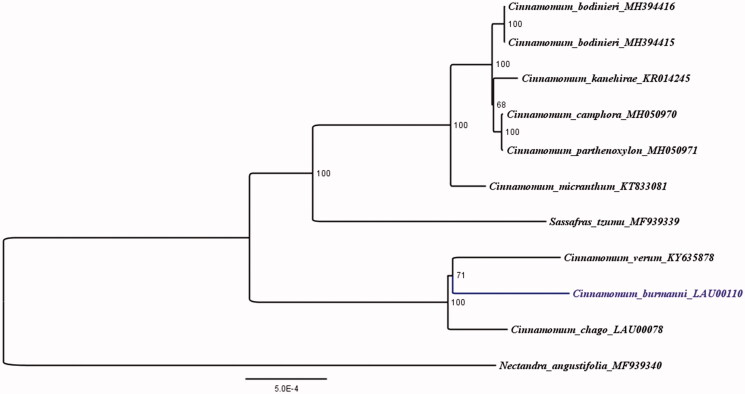
The ML phylogenetic tree for *C. burmanni* based on other 10 species (8 in *Cinnamomum*, 1 in *Nectandra* and 1 in *Sassafras*) plastid genomes.

## Data Availability

The plastome data of the *C. burmanni* will be submitted to Lauraceae Chloroplast Genome Database (https://lcgdb.wordpress.com). Accession numbers are LAU00110.
